# ARID1A Alterations Are Associated with *FGFR3*-Wild Type, Poor-Prognosis, Urothelial Bladder Tumors

**DOI:** 10.1371/journal.pone.0062483

**Published:** 2013-05-01

**Authors:** Cristina Balbás-Martínez, María Rodríguez-Pinilla, Ariel Casanova, Orlando Domínguez, David G. Pisano, Gonzalo Gómez, Josep Lloreta, José A. Lorente, Núria Malats, Francisco X. Real

**Affiliations:** 1 Epithelial Carcinogenesis Group, Molecular Pathology Programme, Spanish National Cancer Research Centre, Madrid, Spain; 2 Lymphoma Group, Molecular Pathology Programme, Spanish National Cancer Research Centre, Madrid, Spain; 3 Genomics Unit, Biotechnology Programme, Spanish National Cancer Research Centre, Madrid, Spain; 4 Bioinformatics Unit, Structural and Computational Biology Programme, Spanish National Cancer Research Centre, Madrid, Spain; 5 Department of Pathology, Hospital del Mar, Barcelona, Spain; 6 Departament de Ciències Experimentals de i la Salut, Universitat Pompeu Fabra, Barcelona, Spain; 7 Urology Service, Hospital del Mar, Barcelona, Spain; 8 Genetic and Molecular Epidemiology Group, Human Cancer Genetics Programme, Spanish National Cancer Research Centre, Madrid, Spain; Biomedical Research Foundation, Academy of Athens, Greece

## Abstract

Urothelial bladder cancer (UBC) is heterogeneous at the clinical, pathological, genetic, and epigenetic levels. Exome sequencing has identified *ARID1A* as a novel tumor suppressor gene coding for a chromatin remodeling protein that is mutated in UBC. Here, we assess ARID1A alterations in two series of patients with UBC. In the first tumor series, we analyze exons 2–20 in 52 primary UBC and find that all mutant tumors belong to the aggressive UBC phenotype (high grade non-muscle invasive and muscle invasive tumors) (*P* = 0.05). In a second series (n = 84), we assess ARID1A expression using immunohistochemistry, a surrogate for mutation analysis, and find that loss of expression increases with higher stage/grade, it is inversely associated with FGFR3 overexpression (*P* = 0.03) but it is not correlated with p53 overexpression (*P* = 0.30). We also analyzed the expression of cytokeratins in the same set of tumor and find, using unsupervised clustering, that tumors with ARID1A loss of expression are generally KRT5/6-low. In this patient series, loss of ARID1A expression is also associated with worse prognosis, likely reflecting the higher prevalence of losses found in tumors of higher stage and grade. The independent findings in these two sets of patients strongly support the notion that *ARID1A* inactivation is a key player in bladder carcinogenesis occurring predominantly in *FGFR3* wild type tumors.

## Introduction

Urothelial carcinoma is the most common type of bladder tumor. Urothelial bladder carcinoma (UBC) constitutes a heterogeneous clinical and pathological entity and subjects presenting with these tumors display a highly variable outcome: approximately 20% of patients with non-muscle invasive bladder cancer (NMIBC) are cured after the first resection whereas 60% undergo multiple lifetime recurrences and 15–20% progress and develop muscle-invasive bladder cancer (MIBC). This important clinical event entails cystectomy and poor prognosis: 50% of patients with MI tumors - either at presentation or during the evolution of the disease - die from the cancer. Patients with low grade (LG) NMIBC rarely progress whereas patients with high grade (HG) NMIBC are at high risk [1]. Pathological grade and stage, multiplicity, and the presence of carcinoma *in situ* are important prognostic factors but there is a need to improve prediction of progression to identify subjects who might benefit from more aggressive early treatment [2].

In agreement with the diverse clinical course, genetic analyses of UBC have revealed a wide heterogeneity [3], [4]. Approximately 60–70% of NMIBC harbor *FGFR3* activating mutations, mainly in association with low grade; *FGFR3* mutant tumors have a good prognosis and a low risk of progression to MIBC [5]–[8]. *PIK3CA* mutations occur in approximately 15% of tumors and tend to be associated with *FGFR3* alterations [9]. *RAS* and *FGFR3* mutations are mutually exclusive, the former occurring in 5–10% of tumors [10]. *FGFR3* wild type tumors include at least two subgroups: those that are of low grade and stage, display papillary growth pattern, and an overall good prognosis, and those that are of high grade and are generally associated with aneuploidy and an aggressive clinical course. In contrast, *Tp53* alterations are associated with HG-NMIBC and with MIBC [3], [4], [6]. Based on current knowledge on the mutation load of human tumors, it is likely that yet unidentified oncogenes - different from *FGFR3*, *PIK3CA,* and *RAS* - and tumor suppressors participate in UBC [11], [12]. There is, therefore, a need to identify additional genes involved in the development of UBC to better understand the relationship between pathological and genetic classifications, and to integrate knowledge on genetic and epigenetic changes [13].

We recently identified a truncating *ARID1A* mutation through UBC exome sequencing and a manuscript recently described the occurrence of *ARID1A* mutations in this tumor [14]. Here, we report that truncating *ARID1A* mutations and loss of expression display an inverse association with *FGFR3* mutations, are independent of p53 alterations, and are mainly associated with poor-prognosis UBC.

## Materials and Methods

### Patients and Tumor Samples

We studied two tumor series. The first, used for *ARID1A* sequencing, comprises 52 UBC cases prospectively recruited between 2009–2011 at Hospital del Mar (Barcelona, Spain) from which fresh tumor DNA was available. The characteristics of patients included in this series are summarized in Table 1. Follow-up for these patients is relatively short. Therefore, we used a second tumor series (n = 84) to assess ARID1A protein expression and its association with outcome. Cases from the latter series were drawn from the Spanish Bladder Cancer/EPICURO Study, comprising patients with incident UBC recruited from 1997–2001 [15], [16]. For all cases, clinical and sociodemographic information was retrieved from hospital records. Tumor staging and grading was carried out according to the TNM classification and the World Health Organization-International Society of Urological Pathology with the two-tiered 2004 WHO redefinition as described [7]. Tumors had previously been classified using a three-tiered system; TaG1 and TaG2 tumors were classified as low-risk because their outcome was very similar [7]. Expert pathologists reviewed diagnostic slides from all tumor blocks from each case to confirm staging/grading and ensure uniformity of classification criteria. Patients from series 2 were prospectively followed-up yearly both through hospital records and by telephone interviews, as described elsewhere [7]. Table 2 summarizes the characteristics of the patients included in this series. Progression was defined as the appearance of a MIBC in a patient having presented with NMIBC or as the development of new tumors in patients treated for primary MIBC. Median follow-up was 62.6 months (range 1–98). All deaths were recorded but only UBC-related deaths (n = 14) were considered for survival analysis. Cases dying from other causes were censored at the time of death for the analysis. Survival was computed as the period comprised between diagnosis and death or last control. All patients provided written informed consent. The Ethics Committee of Institut Municipal d’Assistència Sanitària (Barcelona) approved the study.

**Table 1:** Characteristics of the patients from whom fresh tumor was used for *ARID1A* sequence analysis.

			N (%)	
Number			52	
Age	Mean (SD)		68.7 (11.8)	
Gender	Male		48 (92.3)	
	Female		4 (7.7)	
Stage/Grade	TaG1		12 (23.1)	
	TaG2		7 (13.52)	
	TaG3		9 (17.3)	
	T1G2		6 (11.5)	
	T1G3		10 (19.2)	
	>T2		8 (15.4)	
Tumor size	<3 cm		10 (19.2)	
	≥3 cm		25 (48.1)	
	Unknown		17 (32.7)	
Multiplicity	Single		36 (69.2)	
	Multiple		12 (23.1)	
	Unknown		4 (7.7)	
Treatment	TUR alone	16 (30.8)		
	TUR+endovesical chemo	1 (1.9)		
	TUR+BCG	6 (11.5)		
	Cystectomy	2 (3.8)		
	TUR+BCG+endov chemo	6 (11.5)		
	TUR+cystectomy	3 (5.8)		
	Others	3 (5.8)		
	Missing	15 (28.8)		

**Table 2:** Characteristics of the patients and tumors included in series 2 (tissue microarray).

		N (%)
Number		84
Age	Mean (SD)	66.4 (9.7)
Gender	Male	74 (88.1)
	Female	10 (11.9)
Stage/Grade	TaG1	21 (25.0)
	TaG2	18 (21.4)
	TaG3	7 (8.3)
	T1G3	12 (14.3)
	>T2	26 (31.0)
Tumor size	<3 cm	27 (32.1)
	≥3 cm	17 (20.2)
	Unknown	40 (47.7)
Multiplicity	Single	47 (56.0)
	Multiple	29 (34.5)
	Unknown	8 (9.5)
Treatment	TUR alone	12 (14.4)
	TUR+endovesical chemo	15 (17.6)
	TUR+BCG	30 (35.8)
	Cystectomy	10 (11.9)
	Systemic Chemotherapy	8 (9.6)
	Radiotherapy	5 (5.9)
	Others	4 (4.8)

### *ARID1A* and *FGFR3* Mutational Analysis

*ARID1A* mutational analysis was performed in cases from the first series, essentially as described [17]. Briefly, exons 2–20 were separately PCR-amplified with AccuPrime Taq DNA polymerase High Fidelity (Invitrogen) on DNA from bladder cancer cell lines (RT112, VMCUB-3, MGH-U3, UM-UC-3 and UM-UC-17) and fresh tumor tissue sections containing >60% neoplastic cells. PCR amplimers from each sample were equimolarly pooled and fragmented to a range of 100–300 bp (Covaris S2 shearing instrument). DNA (40–80 ng/sample) was processed through successive enzymatic treatments of end-repair, dA-tailing, and ligation to indexed adapters following the TruSeq DNA sample preparation recommendations (Illumina). Adapter-ligated libraries were amplified by limited-cycle PCR for 10 cycles, subsequently multiplexed and sequenced for 38 cycles on a single read format (Genome Analyzer IIx with SBS TruSeq v5 reagents, Illumina). Sanger sequencing was used to verify *ARID1A* variants identified in the exome sequencing study. Relevant primers can be found in Table S1. *FGFR3* mutational analysis was performed on cases from both series as described elsewhere [7], [18]; we used the SnapSHOT assay [19] for selected cases and verified mutations by Sanger sequencing of PCR products.

### Bioinformatics Analysis

Sequence tags from all samples were independently aligned using Novoalign V2.07.04 (Novocraft, Selangor, Malaysia) versus the genomic *ARID1A* sequence (RefSeq NM_139135) as obtained from UCSC Genome Browser [20] on Human Feb. 2009 (GRCh37/hg19) assembly. Aligned positions were filtered for high quality and processed with a combination of SAMtools and custom Perl scripts. The functional effect of the variants was predicted using SIFT [21].

### Gene Expression Analysis of Publicly Available Datasets

Normalized bladder cancer gene expression data were obtained from NCBI Gene Expression Omnibus (GEO) database for studies GSE89 [22] and GSE32894 [23]. GEPAS 4.0 (http://www.gepas.org/) was used to pre-process the data, obtaining the average values for all probes mapping within a single locus. Three tumor subgroups were defined according to tumor stage/grade and known prevalence of genetic alterations: LG-NMIBC (TaG1, TaG2), HG-NMIBC (TaG3, T1G3), and MIBC (≥T2). The average expression of genes of interest was calculated for each of these groups and values were normalized with respect to those of the best prognosis group. An Anova limma analysis was performed on the pre-processed data using the POMELO online software (http://pomelo2.bioinfo.cnio.es/); differentially expressed genes were subsequently identified through a t-test, using an FDR adjusted P-value <0.5 as threshold of significance.

### Immunohistochemistry (IHC)

Immunohistochemical analyses were performed on tissue microarrays containing cores representative of the corresponding tumor, obtained from formalin-fixed paraffin-embedded tissue blocks from patients included in the second series. The following antibodies were used: ARID1A (2 µg/mL) (3H2, Abnova), FGFR3 (8 µg/mL) (B-9/sc-13121, Santa Cruz), p53 (DO-7, Novocastra), β-CAT (Beta-Catenin-1, Dako, ready-to-use), E-CAD (1∶50) (NCH-38, Dako), P-CAD (1∶75) (56, B.D. Transduction Laboratories), Ki67 (MIB-1, Dako, ready-to-use), KRT5/6 (D5/16B4, Dako, ready-to-use), KRT14 (1∶25) (LL002, Novocastra Laboratories), and KRT20 (Ks20.8, Dako, ready-to-use). Antigen retrieval and IHC were carried out as described elsewhere [18], [24], [25]. Immunoreactivity was scored according to intensity (scale 0–3) and percentage of positive cells (0–100%); IHC score was calculated as the product of intensity and percentage of positive cells. Samples were subjected to unsupervised clustering analysis based on IHC scores using the heatmap.2 function of the gplots package within the R 2.15.1 statistical environment.

### Cell Culture and Functional Assays

SW800, 253J, 639V, VMCUB-3, SW1710, SCaBER, and HEK293 cells were purchased from the American Type Culture Collection (Rockville, MD, US); RT112 [26] and MGH-U3 [27] cells were kindly provided by F. Radvanyi (Institut Curie, Paris, France); UM-UC-6, UM-UC-7, UM-UC-3, and UM-UC-18 were kindly provided by H. B. Grossman (MD Anderson Cancer Center, Houston, TX, US) [28]. All cells were regularly tested to ensure that they were free of Mycoplasma contamination. UBC cells and HEK-293T cells were cultured under standard conditions. Control non-targeting or ARID1A-targeting lentiviral particles were produced in HEK-293T cells using Sigma Mission plasmids following the manufacturer’s instructions. Virus-containing supernatant was collected 24 h later, filtered, and used to infect the corresponding UBC cells in the presence of hexadimethrine bromide polybrene (5 µg/ml) (Sigma); two rounds of infection were performed with a 24 h time interval. Infected cells were selected for 48 h in medium containing puromycin (2 µg/ml) (Sigma). For growth assays, 4×10^3^ puromycin-selected cells were seeded in triplicate in 6-well plates; 4 days later, cells were washed with PBS, fixed in methanol, and incubated with 0.5% crystal violet in 25% methanol. Because the interfered cells did not form compact colonies, counting did not provide an accurate measurement of growth. Crystal violet was eluted in 10% acetic acid and absorbance was quantified at 590 nm using a biophotometer (Eppendorf).

### Immunoblotting

Cells in log-growth phase were collected 48 h after puromycin selection and lysed in RIPA buffer (10 mM Tris–HCl, pH7.5, 1 mM EDTA, 1% Triton X-100, 0.1% SDS, 0.1% Na-deoxycholate, 100 mM NaCl) supplemented with the Complete protease inhibitor (Sigma) and a phosphatase inhibitor (Sigma) cocktails. After sonication, proteins (50 µg) were fractionated by SDS-PAGE using a discontinuous 4% concentrating-6% resolving gel, transferred to a nitrocellulose membrane, and incubated with anti-ARID1A monoclonal antibody (M02, clone 3H14, Abnova) (1∶1000 dilution). After washing, anti-mouse or anti-rabbit horseradish peroxidase-labeled antibodis (Amersham Biosciences) were added. Rabbit anti-Myosin-IIa (Cell Signaling) served as a loading control. Reactions were developed using enhanced chemiluminiscence (Amersham Biosciences).

### RT-qPCR

Total RNA was isolated from cells in log-growth phase using the GenElute Mammalian Total RNA kit (Sigma). Following DNase treatment (DNAfree, Ambion), RNA was reverse-transcribed (Taqman Reverse Transcription Reagents kit, Applied Biosystems) and 20 ng RNA-equivalent were used for RT-qPCR using a 7900H Fast Real Time PCR System (Applied Biosystems). The following primers were used for *ARID1A* mRNA analysis: CCCCTCAATGACCTCCAGTA (forward) and ATCCCTGATGTGCTCACTCC (reverse). All reactions were performed in triplicate, and expression levels were normalized to individual *HPRT* values. **Statistical analyses**. Categorical data were reported by numbers and percentages. Associations between ARID1A mutation/loss of expression and the main characteristics of the patients were assessed using the chi-square test, T test, ANOVA, Mann-Whitney (MW), or Kruskal-Wallis (KW) as appropriate. Associations between markers were evaluated using the chi-square test. Survival data were analysed using Kaplan-Meier curves and the differences between curves were assessed with the log-rank test. Statistical significance was considered at 0.05. R Software (version 2.14, available at http://www.r-project.org/) was used for statistical analysis.

## Results

### *ARID1A* Mutations and Expression in Bladder Cancer

We identified a truncating mutation in *ARID1A* through the initial analysis of 2 bladder cancer exomes. *ARID1A* mutations have been reported to be frequent in ovarian clear cell carcinomas [17], [29] and mutations in genes coding for it and for other components of chromatin remodeling complexes have recently been demonstrated in a wide variety of tumors [14], [30]–[33]. Therefore, we expanded the mutational analysis to a larger tumor panel representative of the UBC spectrum.

**Figure 1:**
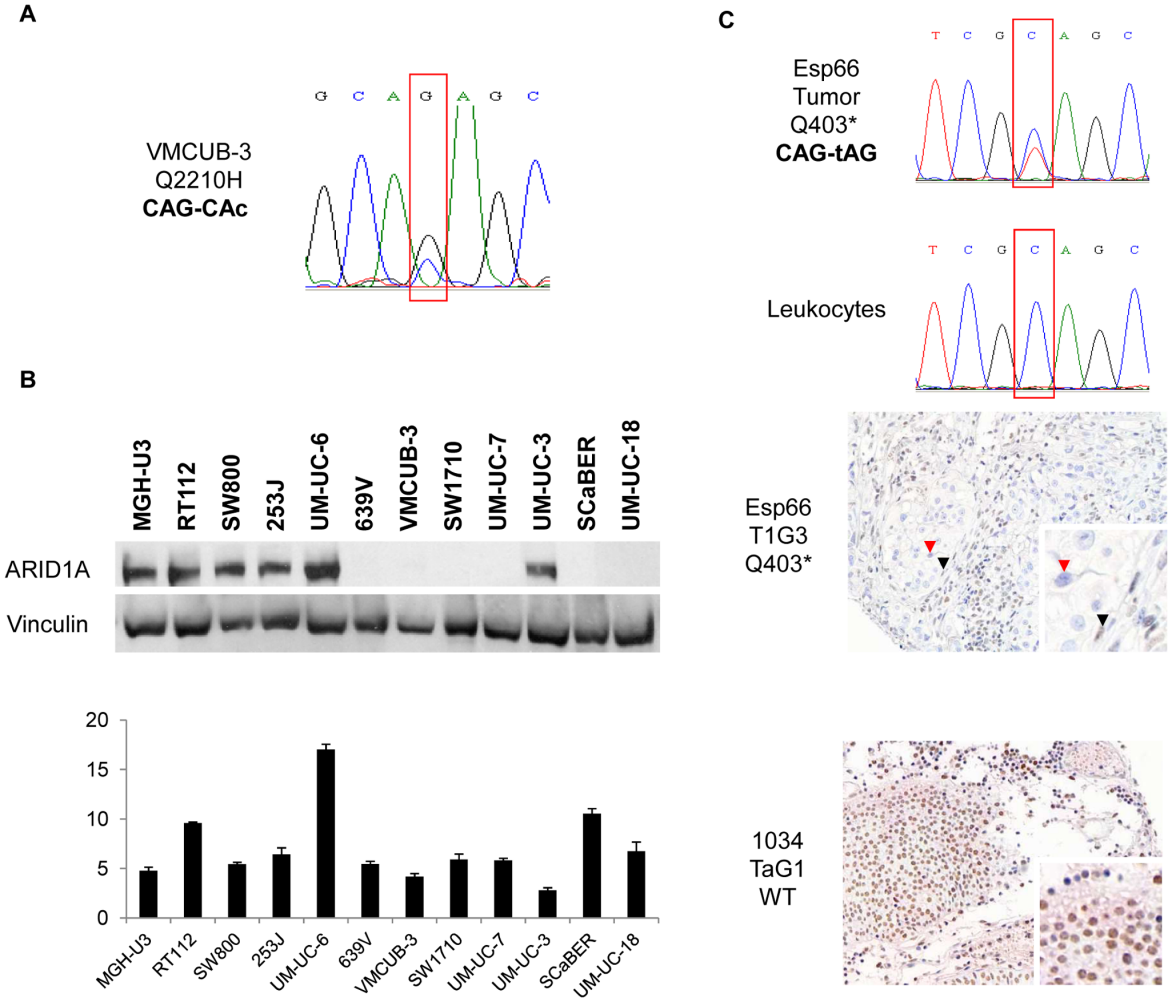
*ARID1A* mutations and expression in UBC. *Panel A*. A G>C transversion identified through Solexa resequencing, confirmed by Sanger sequencing of independent PCR products, leading to a predicted Q2210H substitution in VMCUB-3 cells. *Panel B*. Western blotting analysis in a panel of UBC cell lines identifies a subset with undetectable expression, including VMCUB-3. mRNA expression was analyzed by RT-qPCR; results are shown as values normalized with respect to the housekeeping gene *HPRT*. *Panel C*. A C>T mutation in codon 403, leading to a premature stop codon, was identified in a primary T1G3 tumor. The mutation was absent from matched normal leukocyte DNA. Lack of protein expression in the corresponding tumor tissue was confirmed using immunohistochemistry. The red arrowhead points to a tumor cell lacking ARID1A staining, whereas the black arrowhead indicates a positive stromal cell. For comparison, a TaG1 tumor with wild type *ARID1A* sequence is shown.

Exons 2–20 of *ARID1A* were analyzed in 5 UBC cell lines and 52 primary tumors. Table S2 and Figure S1 show the average depth of reads/exon and the individual sample sequencing breadth and depth, respectively. One cell line (VMCUB-3) and 6 tumors harbored mutations in ≥10% of the reads per given nucleotide position, for a total of 11 single nucleotide variants. There was no relationship between variant allele frequency and sequencing depth (Figure S2A). Four mutations were detected in VMCUB-3, one of which was nonsense (E1733*) and 2 were missense (D1738N and Q2210H) (Figure 1A, Figure S3, Table 3); in addition, a synonymous substitution was identified (L1922L). ARID1A was not detected by western blotting in these cells (Figure 1B). Using a panel of UBC lines, we did not find a good correlation between mRNA and protein expression levels, assessed by RT-qPCR and western blotting (Figure 1B), respectively.

**Table 3:** Summary of mutations found in the *ARID1A* resequencing study.

Sample	Stage	Amino acid substitution	Affected *ARID1A* exon	Variant frequency (%)
Esp21	TaG1	N2066D	20	45.5
Esp66	T1G3	Q403*	2	39.7
Esp69	T3G3	S769*	7	38.1
ISBLAC3800	T1G3	C2052*	20	16.2
ISBLAC3803	T1G3	S571L	3	47.8
		S614*	4	43.5
ISBLAC5559	T1G3	Q393*	2	50
VMCUB-3	Cell line	E1733*	20	18.6
		D1738N	20	21.8
		Q2210H	20	13.9
		L1922L	20	21.5

Non-synonymous mutations found at a frequency ≥10% that were confirmed by Sanger sequencing are shown here.

* denotes a truncating mutation.

Six primary tumors harbored 7 mutations predicted to be damaging (6/52 = 11.5%); 5 of them were nonsense and 2 were missense (N2066D and S571L) (Table 2). One tumor had 2 mutations, one non-sense and one missense. All the mutations were confirmed in independent PCR reactions using Sanger sequencing (Figure 1, Figure S3). The 5 truncating mutations occurred in tumors that were either high grade NMI-BC or MI-BC. One missense mutation was found in a TaG1 tumor. Overall, 0/19 non-aggressive and 5/33 aggressive tumors had a truncating mutation (P = 0.049).

Six additional mutations, predicted to be damaging, were detected at a frequency (<10%) that precluded confirmation by Sanger sequencing; 5 of them led to missense substitutions (Figure S2B).

### *ARID1A* Mutations and *FGFR3* and *Tp53* Alterations in UBC: Relationship with Tumor Aggressiveness

We compared the mutational status of *ARID1A* and *FGFR3* in the 50 tumors for which this information was available: all 5 tumors that had truncating *ARID1A* mutations had *FGFR3* wild type sequences (p = 0.056, Fisher’s exact test), suggesting that the two genes are involved in different genetic pathways. To expand this analysis, we took advantage of the data reported by Gui *et al*. [14] which includes mainy MIBC: in their series 13/97 tumors had a mutation in *ARID1A* and 9/97 tumors had a mutation in *FGFR3*; 1/97 had a mutation in both genes supporting a lack of association between both genetic alterations in this tumor subgroup.

*ARID1A* pathogenic mutations have been reported to be associated with loss of protein expression [17], [31]. We confirmed these observations in the index tumor reported here (Figure 1C). To assess the association of ARID1A expression and clinical-pathological characteristics of tumors, we used an independent series, for which TMAs were available, including 39 LG-NMIBC, 19 HG-NMIBC, and 26 MIBC. ARID1A expression score was significantly lower in more aggressive tumors (ANOVA P = 9.9×10^−6^; KW P = 3×10^−5^), in agreement with the observation that *ARID1A* mutations are more common in this tumor subgroup.

**Figure 2:**
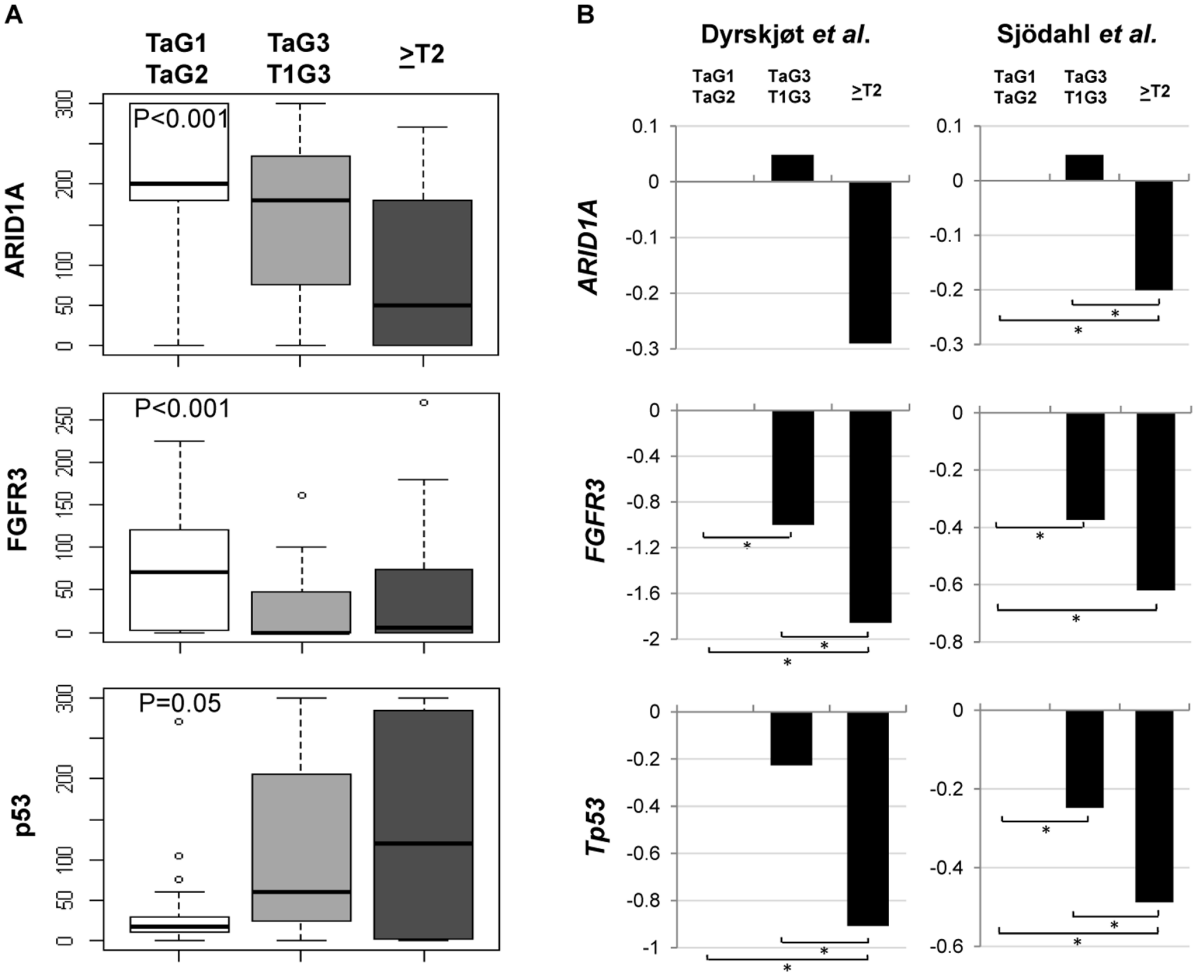
Loss of ARID1A expression is associated with more aggressive UBC. UBC cases were classified in three categories: low grade NMI (TaG1 and TaG2 tumors), high grade NMI (TaG3 and T1G3 tumors), and MI (≥T2 tumors). *Panel A*. ARID1A immunohistochemical score is significantly lower in more aggressive, advanced tumors. FGFR3 immunohistochemical score, which is directly associated with *FGFR3* mutations, is also significantly lower in more aggressive tumors. By contrast, p53 score is higher in more aggressive tumors. *Panel B*. Differential expression of *ARID1A, FGFR3* and *TP53* at the mRNA level is observed in two different, independent UBC microarray series: the mRNA levels of all 3 genes are significantly lower in MIBC. *denotes an FDR adjusted *P*-value <0.5.

Immunohistochemistry was used as a surrogate to assess the status of two of the main genes involved in UBC: *FGFR3* activating mutations are generally associated with *FGFR3* mRNA and protein overexpression [34] and *Tp53* inactivation is generally associated with p53 nuclear overexpression [18], [35], [36]. As expected, FGFR3 immunohistochemical scores were significantly associated with *FGFR3* mutations (ANOVA P = 3×10^−5^, KW P = 3×10^−4^) and were higher in the low grade NMIBC than in the more aggressive tumor groups of high grade NMIBC and MIBC (ANOVA P = 0.038, KW P = 0.026). In contrast, p53 nuclear overexpression was increasingly higher with increasing tumor stage/grade, reflecting protein accumulation associated with *Tp53* mutations (ANOVA P = 0.05, KW P = 0.32). ARID1A scores were significantly correlated with FGFR3 expression scores (Spearman correlation P = 0.03) but not with p53 scores (p = 0.30) (Figure 2A).

To determine whether *ARID1A* and *FGFR3* are differentially expressed in the three UBC subgroups at the RNA level, we analyzed two independent public UBC expression datasets and confirmed that both *ARID1A* and *FGFR3* mRNA expression levels are significantly lower in MI-UBC, in agreement with the fact that *FGFR3* mutations are associated with *FGFR3* mRNA overexpression and are less frequent in aggressive tumors (Figure 2B). Similarly, *TP53* mRNA expression levels were significantly lower in MIBC, possibly as a result of gene losses.

### ARID1A Expression and Cell Differentiation Markers

**Figure 3:**
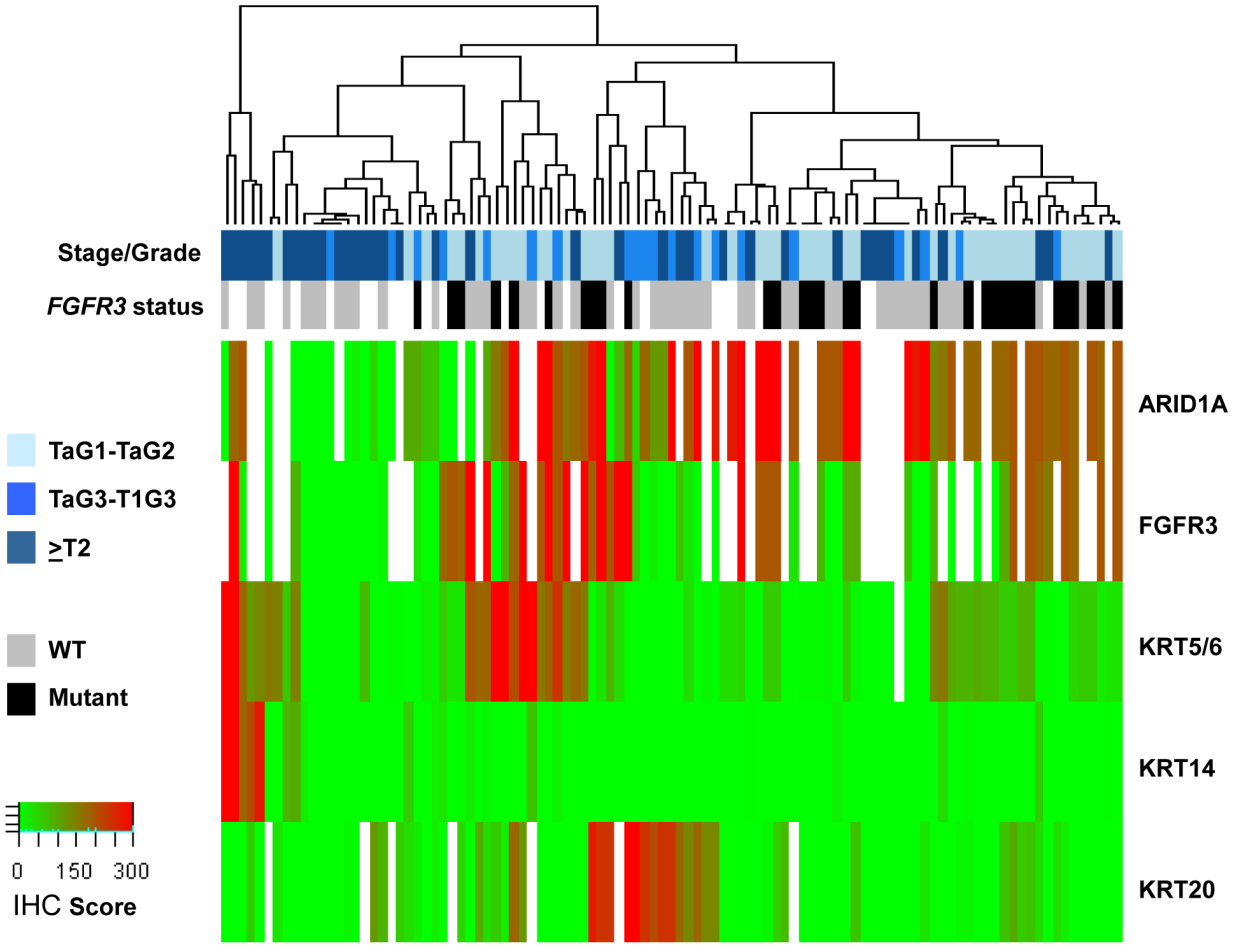
Relationship between ARID1A and cell differentiation markers, as detected using immunohistochemistry in tumor tissue microarrays. UBC cases were classified in three categories: LG-NMIBC (TaG1 and TaG2 tumors), HG-NMIBC (TaG3 and T1G3 tumors), and MI (≥T2 tumors). Non-hierarchical clustering of IHC scores for ARID1A, FGFR3, KRT5/6, KRT14, and KRT20 was performed. IHC scores are shown in a green-red color code. Color bars below the dendogram include information about tumor stage and grade (tones of blue) and *FGFR3* mutational status (grey/black) when known. White squares indicate that information for that parameter is not available.

We compared expression of ARID1A and a set of urothelial differentiation markers [37] using IHC and performed unsupervised clustering of the samples (Figure 3 and Figure S4). This analysis confirmed that tumors expressing low levels of ARID1A generally exhibited low levels of FGFR3. Tumors showing low levels of ARID1A also tended to display low expression of KRT5/6 and KRT20 (Figure 3).

### ARID1A Expression in Tumors and Patient Outcome

**Figure 4:**
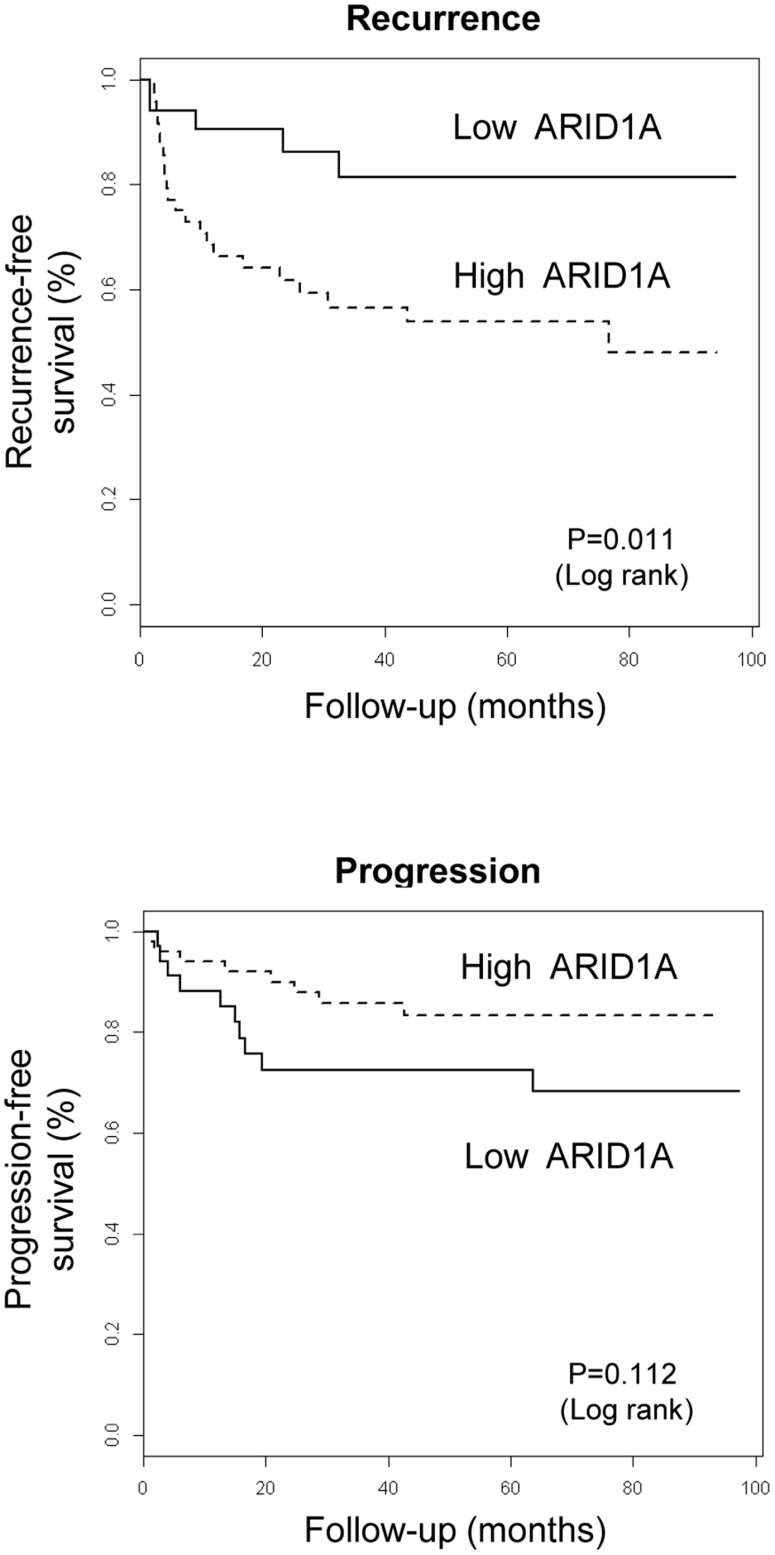
Loss of ARID1A expression is associated with more aggressive UBC and with patient outcome. ARID1A expression was assessed by IHC on tissue microarrays. Patients (n = 84) were followed-up as indicated in Methods and classified as having “recurred”, “progressed”, or being free of disease. Patients with high ARID1A-expresssing tumors display a lower risk of recurrence and a higher risk of progression indicating a more aggressive clinical course.

Kaplan-Meier analysis showed that patients with tumors showing low ARID1A expression (IHC score<180) showed a significantly lower rate of tumor recurrence (P = 0.011) but had a higher rate of tumor progression (P = 0.112), further indicating that loss of ARID1A expression is associated with more aggressive tumors (Figure 4).

### Functional Analysis of ARID1A in Cultured UBC

To assess the role of ARID1A in UBC, we knocked it down in cultured UBC lines. Six of 12 UBC lines analyzed lacked ARID1A expression, including VMCUB-3 (Figure 1B).

**Figure 5:**
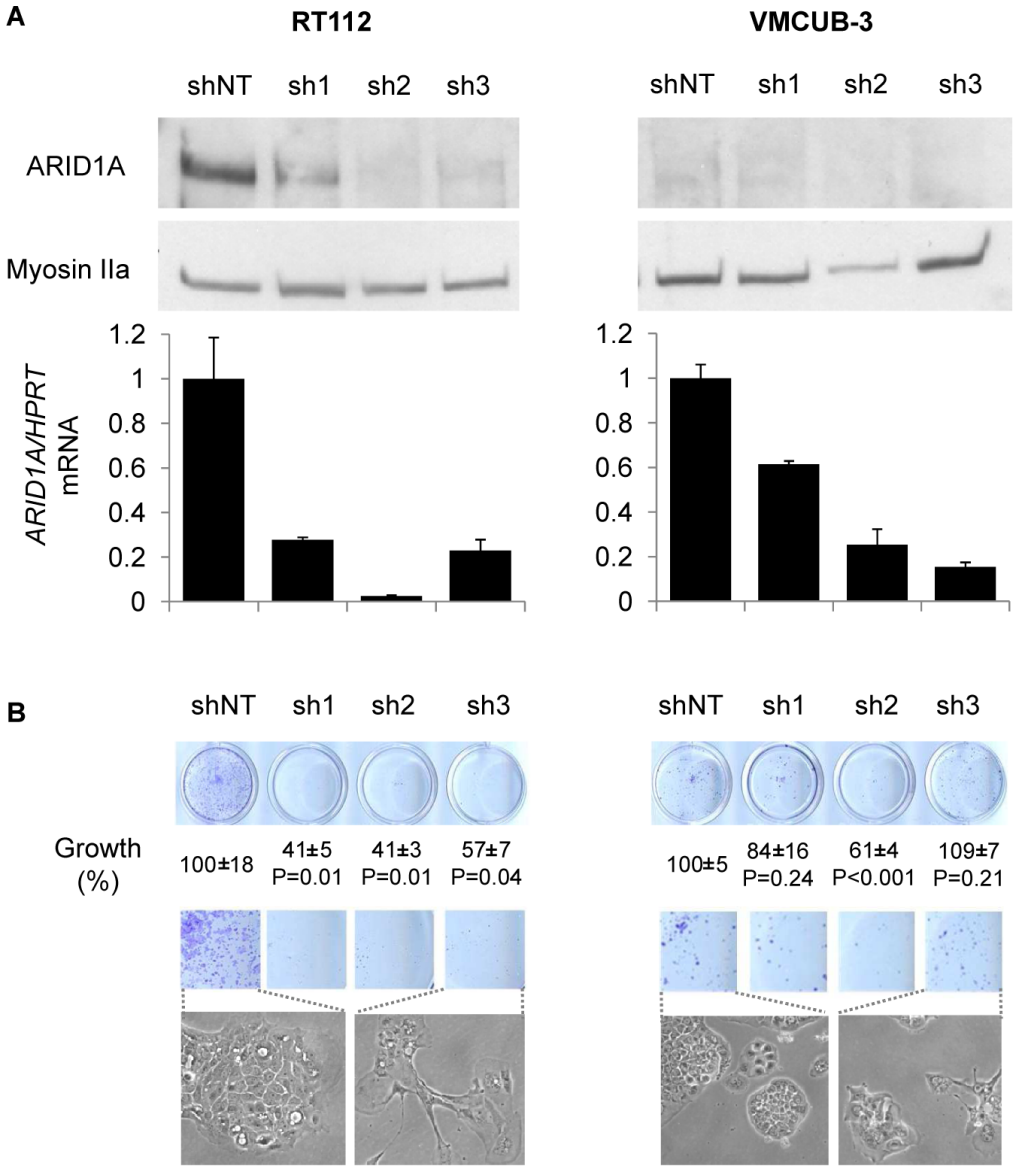
Effects of *ARID1A* knockdown in UBC cell lines. *Panel A*. *ARID1A* was knocked-down using three different shRNAs in the RT112 and VMCUB-3 cells. The knock-down was efficient at both the protein and mRNA levels. The bars represent the relative quantification of ARID1A mRNA levels taking non-targeting shRNA interfered cells as controls. *Panel B*. The quantification colony formation is shown, with error intervals of results from triplicate experiments denoting SEM. In RT112 cells, ARID1A knockdown was associated with reduced colony formation. By contrast, no major effects were observed in VMCUB-3 cells harboring an *ARID1A* mutation. Representative morphological changes in cells interfered with control shNT (scrambled shRNA) and with one of the shRNAs targeting *ARID1A* are shown.

RT112 cells express high levels of active wild type FGFR3, lack *ARID1A* mutations, are wild type for *Tp53* [38], and show features reminiscent of low grade NMIBC. Upon lentiviral knockdown, a significant 50% reduction in cell growth was observed and cells displayed a flatter morphology (Figure 5). These effects were consistently observed with 3 different shRNAs in 3 independent experiments. Similar results were obtained in 253J cells, which also express ARID1A by western blotting (Figure S5). By contrast, knockdown with the same lentiviruses in VMCUB-3 - lacking ARID1A expression and with mutant *Tp53* - had no consistent effects on colony formation (Figure 5). The strong inhibition of cell viability upon ARID1A knockdown did not allow performing additional functional studies such as cell migration or invasion.

## Discussion

Proteins involved in chromatin remodeling and histone post-translational modifications are emerging as powerful tumor suppressors inactivated in a wide variety of human cancers [32]. The list of tumor types in which *ARID1A* mutations have been identified keeps expanding [39]–[41] and increasing evidence indicates that epigenetic silencing can also contribute to its functional inactivation [42], [43].

In this work we focused on the analysis of *ARID1A* mutations and loss of expression and placed them in the context of current knowledge of UBC molecular pathogenesis. By analyzing a broad range of tumors, representative of UBC diversity, we find that *ARID1A* alterations generally occur in *FGFR3* wild type, poor-prognosis bladder tumors. We find that 11.5% of all UBC harbor *ARID1A* pathogenic mutations, most of them truncating, and that multiple gene mutations can be found in some of the tumors/cell lines, supporting biallelic inactivation or intratumoral heterogeneity, as has been reported in other tumor types [44]. Because the study of Gui *et al.* [14] focused almost exclusively on MIBC, the relationship between *ARID1A* and *FGFR3* mutations could not be assessed fully in that series. The more representative basis of the samples analyzed here, both at the mutational and protein expression levels, has allowed us to identify an inverse association between *ARID1A* and *FGFR3* mutations and the predominance of *ARID1A* mutations in more aggressive tumors. Interestingly, both the study of Gui *et al.* and a more recent report [45] indicate that *ARID1A* mutations contribute mainly to a subset of poor-prognosis *TP53* wild type tumors, suggesting that the effects of both genes converge functionally. In the last year, several reports in other tumor types have analyzed whether *ARID1A* alterations are restricted to selected genetic pathways in specific tumor types: in gastric cancer, *ARID1A* mutations are also associated with *TP53* wild type tumors [46] and in ovarian clear-cell carcinoma *ARID1A* alterations are reported to be an early event associated with *PIK3CA* mutations [47], [48].

The mechanisms through which chromatin remodelers contribute to neoplastic transformation are not fully understood [32], [33], [49]. However, there is evidence supporting a role in the regulation of cell proliferation and differentiation. For example, ARID1A has been shown to regulate the expression of genes within the c-Myc programme and BRG1, another protein of the SWI/SNF complex, antagonizes Myc activity and favors cell differentiation through binding to the promoter of its targets [50]. We therefore analyzed whether ARID1A loss of expression is associated with epithelial differentiation markers in UBC that have - in turn -been related to the genetic pathways involved in this tumor [23]. Using a TMA containing samples covering the full spectrum of the disease, we found that the cluster of ARID1A-negative tumors exhibits low expression levels of FGFR3 and cases with low ARID1A expression were generally KRT5/6 and KRT20-negative. Larger patient series are required to confirm these findings and fully understand their biological and clinical significance. The observation that the ARID1A-negative group is similar to the genomically unstable tumor subgroup of Sjödahl [23] suggests that ARID1A might contribute to the maintenance of genomic stability, possibly through its role in chromatin remodeling. It is also tempting to speculate that alterations in *ARID1A*, and possibly in genes coding for other chromatin remodelers or histone modifiers, might contribute to the multiple regional epigenetic silencing phenotype reported to occur in *FGFR3*-wild type UBC [51]. The association of ARID1A loss of expression with patient prognosis reported here may be secondary to the higher prevalence of alterations in tumors of more advanced stage and higher grade or to genomic instability, among others. To address these issues will require both functional studies and the analysis of larger patient series.

To address whether ARID1A plays a role in the control of cell proliferation and differentiation, we knocked it down in UBC lines expressing the protein. Surprisingly, we found that, upon efficient protein down-regulation, cells showed reduced viability and colony formation capacity that hampered further analysis of other phenotypic properties. This effect is at odds with its role as a tumor suppressor. There is a paucity of information regarding the mechanisms through which ARID1A loss-of-function acts in cancer cells and there are conflicting reports regarding the effects of its inactivation: in ovarian cancer, a moderate knockdown was associated with increased proliferation [52]. By contrast, in pancreatic cancer - where *ARID1A* mutations and loss of expression are also common [53] - *in vitro* knockdown led to divergent effects depending on the cell type used, including reduced cell proliferation [54] as shown here for RT112 cells. Context-specific effects or dose-related differences may account for these discrepancies. In mice, inactivation of one *Arid1a* allele is embryonic lethal, supporting that cells can be exquisitively sensitive to changes in protein dose [55]. Further work is required to assess the mechanisms through which ARID1A inactivation favors tumor progression, to determine how it modulates the effects of oncogenes or tumor suppressors, and whether different mutations have distinct biological effects.

We conclude that *ARID1A* mutations and loss of expression play an important role in UBC development and are associated with a more aggressive pathway of genetic progression.

## Supporting Information

Figure S1 **Resequencing data metrics.** *Panel A.* Average sequencing breadth of reads per exon for each sample. *Panel B.* Average sequencing depth of reads per exon for each sample.
(TIF)

Figure S2 **Mutation occurrence and frequency.** *Panel A.* SNV frequency plotted against sequencing depth. *Panel B.* SIFT predictions for mutations comparing findings occurring for variants called at high vs. low frequency (threshold at 10%).
(TIF)

Figure S3 **Sanger sequencing verification of all mutations detected in the resequencing study.** All mutations detected at a frequency >10% were verified in both tumors and VMCUB-3 cells. The wild type sequence in normal leukocyte DNA is also shown for selected cases.
(TIF)

Figure S4 **Relationship between ARID1A levels and those of other well-established UBC markers.** UBC cases were classified in three categories: low grade NMI (TaG1 and TaG2 tumors), high grade NMI (TaG3 and T1G3 tumors), and MI (>T2 tumors). Nonhierarchical clustering of IHC scores for ARID1A, FGFR3, KRT5/6, KRT14, KRT20, β-CAT, Ki67, ECAD, and P-CAD was performed. IHC scores are shown using a green-red color code. Color bars under the dendogram include information about prognosis (pistachio/bourbon), tumor stage and grade (tones of blue), and FGFR3 mutational status (grey/black) when known. White squares indicate that information for that parameter is not available.
(TIF)

Figure S5 **Effects of ARID1A knockdown in the 253J UBC cells.** *Panel A.* ARID1A was knocked-down using three different shRNAs in the 253J cells. The knock-down was efficient at both the protein and mRNA levels. The bars represent the relative quantification of ARID1A mRNA levels taking non-targeting shRNA interfered cells as controls. *Panel B.* The quantification colony formation is shown, with error intervals of results from triplicate experiments denoting SEM. ARID1A knockdown was associated with reduced colony formation. Representative morphology changes in the cultured cells interfered for shNT (scrambled shRNA) and one of the shRNAs targeting ARID1A are shown.
(TIF)

Table S1 **List of primers used for ARID1A resequencing and Sanger sequencing.**
(TIF)

Table S2 **Summary of reads per exon in the ARID1A resequencing study.** Exon starting and ending positions are shown, along with the exon length in base pairs and the average sequencing depth.
(TIF)

## References

[1] Sylvester RJ, van der Meijden AP, Oosterlinck W, Witjes JA, Bouffioux C, et al (2006) Predicting recurrence and progression in individual patients with stage Ta T1 bladder cancer using EORTC risk tables: a combined analysis of 2596 patients from seven EORTC trials. Eur Urol 49: 466–475. 16442208 10.1016/j.eururo.2005.12.031

[2] Thomas F, Rosario DJ, Rubin N, Goepel JR, Abbod MF, et al.. (2012) The long-term outcome of treated high-risk nonmuscle-invasive bladder cancer: Time to change treatment paradigm? Cancer; doi: 10.1002/cncr.27587. [Epub ahead of print] PubMed PMID: 22544645. 10.1002/cncr.27587 22544645

[3] Wu XR (2005) Urothelial tumorigenesis: a tale of divergent pathways. Nat Rev Cancer 5: 713–725. 16110317 10.1038/nrc1697

[4] Luis NM, López-Knowles E, Real FX (2007) Molecular biology of bladder cancer. Clin Transl Oncol 9: 5–12. 17272224 10.1007/s12094-007-0003-x

[5] Cappellen D, De Oliveira C, Ricol D, de Medina S, Bourdin J, et al (1999) Frequent activating mutations of FGFR3 in human bladder and cervix carcinomas. Nat Genet 23: 18–20. 10471491 10.1038/12615

[6] Bakkar AA, Wallerand H, Radvanyi F, Lahaye JB, Pissard S, et al (2003) FGFR3 and TP53 gene mutations define two distinct pathways in urothelial cell carcinoma of the bladder. Cancer Res 63: 8108–8112. 14678961

[7] Hernández S, López-Knowles E, Lloreta J, Kogevinas M, Amorós A, et al (2006) Prospective study of FGFR3 mutations as a prognostic factor in nonmuscle invasive urothelial bladder carcinomas. J Clin Oncol 24: 3664–3671. 16877735 10.1200/JCO.2005.05.1771

[8] van Rhijn BW, van der Kwast TH, Liu L, Fleshner NE, Bostrom PJ, et al (2012) The FGFR3 mutation is related to favorable pT1 bladder cancer. J Urol 187: 310–314. 22099989 10.1016/j.juro.2011.09.008

[9] López-Knowles E, Hernández S, Malats N, Kogevinas M, Lloreta J, et al (2006) PIK3CA mutations are an early genetic alteration associated with FGFR3 mutations in superficial papillary bladder tumors. Cancer Res 66: 7401–7404. 16885334 10.1158/0008-5472.CAN-06-1182

[10] Jebar AH, Hurst CD, Tomlinson DC, Johnston C, Taylor CF, et al (2005) FGFR3 and Ras gene mutations are mutually exclusive genetic events in urothelial cell carcinoma. Oncogene 24: 5218–5225. 15897885 10.1038/sj.onc.1208705

[11] Wong KM, Hudson TJ, McPherson JD (2011) Unraveling the genetics of cancer: genome sequencing and beyond. Annu Rev Genomics Hum Genet 12: 407–430. 21639794 10.1146/annurev-genom-082509-141532

[12] Zhang J, Baran J, Cros A, Guberman JM, Haider S, et al.. (2011) International Cancer Genome Consortium Data Portal–a one-stop shop for cancer genomics data. Database (Oxford) bar026. 10.1093/database/bar026 PMC3263593 21930502

[13] Lindgren D, Sjödahl G, Lauss M, Staaf J, Chebil G, et al (2012) Integrated genomic and gene expression profiling identifies two major genomic circuits in urothelial carcinoma. PLoS One 7: e38863. 22685613 10.1371/journal.pone.0038863 PMC3369837

[14] Gui Y, Guo G, Huang Y, Hu X, Tang A, et al (2011) Frequent mutations of chromatin remodeling genes in transitional cell carcinoma of the bladder. Nat Genet 43: 875–878. 21822268 10.1038/ng.907 PMC5373841

[15] García-Closas M, Malats N, Silverman D, Dosemeci M, Kogevinas M, et al (2005) NAT2 slow acetylation and GSTM1 null genotypes increase bladder cancer risk: results from the Spanish Bladder Cancer Study and meta-analyses. Lancet 366: 649–659. 16112301 10.1016/S0140-6736(05)67137-1 PMC1459966

[16] Guey LT, García-Closas M, Murta-Nascimento C, Lloreta J, Palencia L, et al (2010) Genetic susceptibility to distinct bladder cancer subphenotypes. Eur Urol 57: 283–292. 19692168 10.1016/j.eururo.2009.08.001 PMC3220186

[17] Wiegand KC, Shah SP, Al-Agha OM, Zhao Y, Tse K, et al (2010) ARID1A mutations in endometriosis-associated ovarian carcinomas. N Engl J Med 363: 1532–1543. 20942669 10.1056/NEJMoa1008433 PMC2976679

[18] Hernandez S, Lopez-Knowles E, Lloreta J, Kogevinas M, Jaramillo R, et al (2005) FGFR3 and Tp53 mutations in T1G3 transitional bladder carcinomas: independent distribution and lack of association with prognosis. Clin Cancer Res 11: 5444–5450. 16061860 10.1158/1078-0432.CCR-05-0122

[19] Hafner C, van Oers JM, Vogt T, Landthaler M, Stoehr R, et al (2006) Mosaicism of activating FGFR3 mutations in human skin causes epidermal nevi. J Clin Invest 116: 2201–2207. 16841094 10.1172/JCI28163 PMC1501112

[20] Fujita PA, Rhead B, Zweig AS, Hinrichs AS, Karolchik D, et al (2010) The UCSC Genome Browser database: update 2011. Nucleic Acids Res 39: D876–D882. 20959295 10.1093/nar/gkq963 PMC3242726

[21] Kumar P, Henikoff S, Ng PC (2009) Predicting the effects of coding non-synonymous variants on protein function using the SIFT algorithm. Nat Protoc 4: 1073–1081. 19561590 10.1038/nprot.2009.86

[22] Dyrskjøt L, Thykjaer T, Kruhøffer M, Jensen JL, Marcussen N, et al (2003) Identifying distinct classes of bladder carcinoma using microarrays. Nat Genet 33: 90–96. 12469123 10.1038/ng1061

[23] Sjödahl G, Lauss M, Lövgren K, Chebil G, Gudjonsson S, et al (2012) A molecular taxonomy for urothelial carcinoma. Clin Cancer Res 18: 3377–3386. 22553347 10.1158/1078-0432.CCR-12-0077-T

[24] Amaral AFS, Méndez-Pertuz M, Muñoz A, Silverman DT, Allory Y, et al (2012) Plasma 25-hydroxyvitamin D3 levels and bladder cancer risk according to tumor stage and FGFR3 status: a mechanism-based epidemiological study. J Natl Cancer Inst 104: 1897–1904. 23108201 10.1093/jnci/djs444 PMC3525815

[25] López-Knowles E, Hernández S, Kogevinas M, Lloreta J, Amorós A, et al (2006) The p53 pathway and outcome among patients with T1G3 bladder tumors. Clin Cancer Res 12: 6029–6036. 17062677 10.1158/1078-0432.CCR-06-0206

[26] Masters JR, Hepburn PJ, Walker L, Highman WJ, Trejdosiewicz LK, et al (1986) Tissue culture model of transitional cell carcinoma: characterization of twenty-two human urothelial cell lines. Cancer Res 46: 3630–3636. 3708594

[27] Lin CW, Lin JC, Prout GR (1985) Establishment and characterization of four human bladder tumor cell lines and sublines with different degrees of malignancy. Cancer Res 45: 5070–5079. 4027986

[28] Sabichi A, Keyhani A, Tanaka N, Delacerda J, Lee IL, et al (2006) Characterization of a panel of cell lines derived from urothelial neoplasms: genetic alterations, growth in vivo and the relationship of adenoviral mediated gene transfer to coxsackie adenovirus receptor expression. J Urol 175: 1133–1137. 16469639 10.1016/S0022-5347(05)00323-X

[29] Jones S, Wang TL, Shih IeM, Mao TL, Nakayama K, et al (2010) Frequent mutations of chromatin remodeling gene ARID1A in ovarian clear cell carcinoma. Science 330: 228–231. 20826764 10.1126/science.1196333 PMC3076894

[30] Jones S, Li M, Parsons DW, Zhang X, Wesseling J, et al (2012) Somatic mutations in the chromatin remodeling gene ARID1A occur in several tumor types. Hum Mutat 33: 100–103. 22009941 10.1002/humu.21633 PMC3240719

[31] Guan B, Mao TL, Panuganti PK, Kuhn E, Kurman RJ, et al (2011) Mutation and loss of expression of ARID1A in uterine low-grade endometrioid carcinoma. Am J Surg Pathol 35: 625–632. 21412130 10.1097/PAS.0b013e318212782a PMC3077471

[32] Wilson BG, Roberts CW (2011) SWI/SNF nucleosome remodellers and cancer. Nat Rev Cancer 11: 481–492. 21654818 10.1038/nrc3068

[33] Guichard C, Amaddeo G, Imbeaud S, Ladeiro Y, Pelletier L, et al (2012) Integrated analysis of somatic mutations and focal copy-number changes identifies key genes and pathways in hepatocellular carcinoma. Nat Genet 44: 694–698. 22561517 10.1038/ng.2256 PMC3819251

[34] Tomlinson DC, Baldo O, Harnden P, Knowles MA (2007) FGFR3 protein expression and its relationship to mutation status and prognostic variables in bladder cancer. J Pathol 213: 91–98. 17668422 10.1002/path.2207 PMC2443273

[35] Malats N, Bustos A, Nascimento CM, Fernandez F, Rivas M, et al (2005) P53 as a prognostic marker for bladder cancer: A meta-analysis and review. Lancet Oncol 6: 678–686. 16129368 10.1016/S1470-2045(05)70315-6

[36] Real FX (2007) p53: it has it all, but will it make to the clinic as a marker in bladder cancer? J Clin Oncol 25: 5341–5344. 18048811 10.1200/JCO.2007.13.1904

[37] Volkmer JP, Sahoo D, Chin RK, Ho PL, Tang C, et al (2012) Three differentiation states risk-stratify bladder cancer into distinct subtypes. Proc Natl Acad Sci USA 109: 2078–2083. 22308455 10.1073/pnas.1120605109 PMC3277552

[38] Rieger KM, Little AR, Swart JM, Kastrinakis WV, Fitzgerald JM, et al (1995) Human bladder carcinoma cell lines as indicators of oncogenic change relevant to urothelial neoplastic progression. Br J Cancer 72: 683–690. 7669581 10.1038/bjc.1995.394 PMC2033904

[39] Cancer Genome Atlas Network (2012) Comprehensive molecular characterization of human colon and rectal cancer. Nature 487: 330–337. 22810696 10.1038/nature11252 PMC3401966

[40] Imielinski M, Berger AH, Hammerman PS, Hernandez B, Pugh TJ, et al (2012) Mapping the hallmarks of lung adenocarcinoma with massively parallel sequencing. Cell 150: 1107–1120. 22980975 10.1016/j.cell.2012.08.029 PMC3557932

[41] Love C, Sun Z, Jima D, Li G, Zhang J, et al (2012) The genetic landscape of mutations in Burkitt lymphoma. Nat Genet 44: 1321–1325. 10.1038/ng.2468 PMC3674561 23143597

[42] Mamo A, Cavallone L, Tuzmen S, Chabot C, Ferrario C, et al (2012) An integrated genomic approach identifies ARID1A as a candidate tumor-suppressor gene in breast cancer. Oncogene 31: 2090–2100. 21892209 10.1038/onc.2011.386

[43] Zhang X, Sun Q, Shan M, Niu M, Liu T, et al (2013) Promoter Hypermethylation of ARID1A Gene Is Responsible for Its Low mRNA Expression in Many Invasive Breast Cancers. PLoS One 8: e53931. 23349767 10.1371/journal.pone.0053931 PMC3549982

[44] Huang J, Deng Q, Wang Q, Li KY, Dai JH, et al (2012) Exome sequencing of hepatitis B virus-associated hepatocellular carcinoma. Nat Genet 44: 1117–1121. 22922871 10.1038/ng.2391

[45] Iyer G, Hanrahan AJ, Milowsky MI, Al-Ahmadie H, Scott SN, et al (2012) Genome sequencing identifies a basis for everolimus sensitivity. Science 338: 221. 22923433 10.1126/science.1226344 PMC3633467

[46] Wang K, Kan J, Yuen ST, Shi ST, Chu KM, et al (2011) Exome sequencing identifies frequent mutation of ARID1A in molecular subtypes of gastric cancer. Nat Genet 43: 1219–1223. 22037554 10.1038/ng.982

[47] Yamamoto S, Tsuda H, Takano M, Tamai S, Matsubara O (2012) Loss of ARID1A protein expression occurs as an early event in ovarian clear-cell carcinoma development and frequently coexists with PIK3CA mutations. Mod Pathol 25: 615–624. 22157930 10.1038/modpathol.2011.189

[48] Yamamoto S, Tsuda H, Takano M, Tamai S, Matsubara O (2012) PIK3CA mutations and loss of ARID1A protein expression are early events in the development of cystic ovarian clear cell adenocarcinoma. Virchows Arch 460: 77–87. 22120431 10.1007/s00428-011-1169-8

[49] Deem AK, Li X, Tyler JK (2012) Epigenetic regulation of genomic integrity. Chromosoma 121: 131–151. 22249206 10.1007/s00412-011-0358-1 PMC3982914

[50] Romero OA, Setien F, John S, Gimenez-Xavier P, Gómez-López G, et al (2012) The tumour suppressor and chromatin-remodelling factor BRG1 antagonizes Myc activity and promotes cell differentiation in human cancer. EMBO Mol Med 4: 603–616. 22407764 10.1002/emmm.201200236 PMC3407948

[51] Vallot C, Stransky N, Bernard-Pierrot I, Hérault A, Zucman-Rossi J, et al (2011) A novel epigenetic phenotype associated with the most aggressive pathway of bladder tumor progression. J Natl Cancer Inst 103: 47–60. 21173382 10.1093/jnci/djq470 PMC3014990

[52] Guan B, Wang TL, ShihIe M (2011) ARID1A, a factor that promotes formation of SWI/SNF-mediated chromatin remodeling, is a tumor suppressor in gynecologic cancers. Cancer Res 71: 6718–6727. 21900401 10.1158/0008-5472.CAN-11-1562 PMC3206175

[53] Birnbaum DJ, Adélaïde J, Mamessier E, Finetti P, Lagarde A, et al (2011) Genome profiling of pancreatic adenocarcinoma. Genes Chromosomes Cancer 50: 456–465. 21412932 10.1002/gcc.20870

[54] Shain AH, Giacomini CP, Matsukuma K, Karikari CA, Bashyam MD, et al (2012) Convergent structural alterations define SWItch/Sucrose NonFermentable (SWI/SNF) chromatin remodeler as a central tumor suppressive complex in pancreatic cancer. Proc Natl Acad Sci USA 109: E252–259. 22233809 10.1073/pnas.1114817109 PMC3277150

[55] Gao X, Tate P, Hu P, Tjian R, Skarnes WC, et al (2008) ES cell pluripotency and germ-layer formation require the SWI/SNF chromatin remodeling component BAF250a. Proc Natl Acad Sci USA 105: 6656–6661. 18448678 10.1073/pnas.0801802105 PMC2373334

