# Correction: ARID1A Alterations Are Associated with *FGFR3*-Wild Type, Poor-Prognosis, Urothelial Bladder Tumors

**DOI:** 10.1371/annotation/6e83489b-e1ff-4523-9b31-08c90fa39030

**Published:** 2014-01-08

**Authors:** Cristina Balbás-Martínez, María Rodríguez-Pinilla, Ariel Casanova, Orlando Domínguez, David G. Pisano, Gonzalo Gómez, Josep Lloreta, José A. Lorente, Núria Malats, Francisco X. Real

There is an error in Figure S3. The correct mutations for Sample ISBLAC3803 are S571L TCG>TTG and S614* TCA>TGA. Please view the corrected Figure S3 here: 
