# Supplementary figures and images for: ARID1A Alterations Are Associated with FGFR3-Wild Type, Poor-Prognosis, Urothelial Bladder Tumors
Source: PLoS One. 2013 May 1;8(5):e62483. doi: 10.1371/journal.pone.0062483 (PMC3641081; doi:10.1371/journal.pone.0062483)

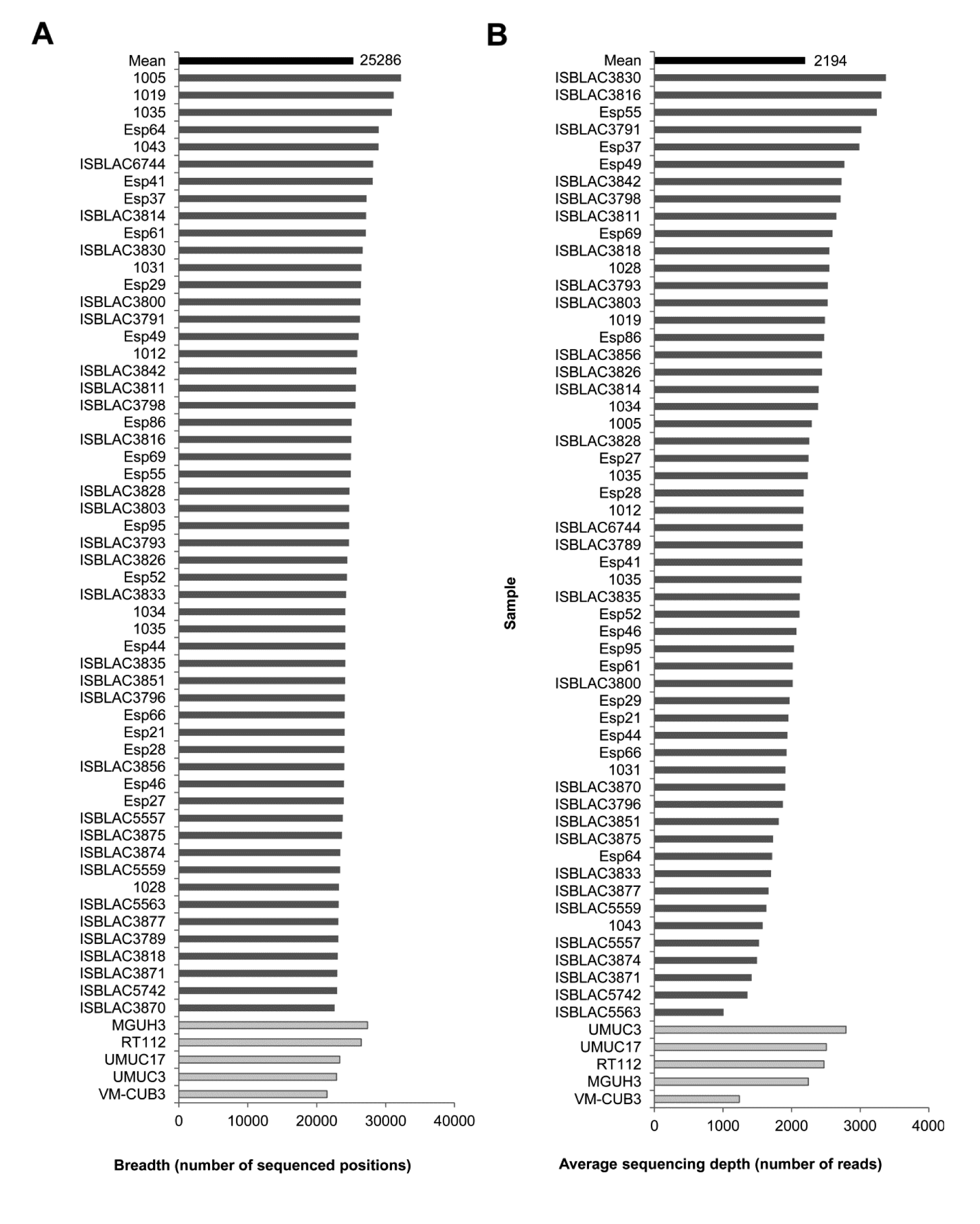

Supplement: Figure S1 — Resequencing data metrics. Panel A. Average sequencing breadth of reads per exon for each sample. Panel B. Average sequencing depth of reads per exon for each sample. (TIF) [file pone.0062483.s001.tif]

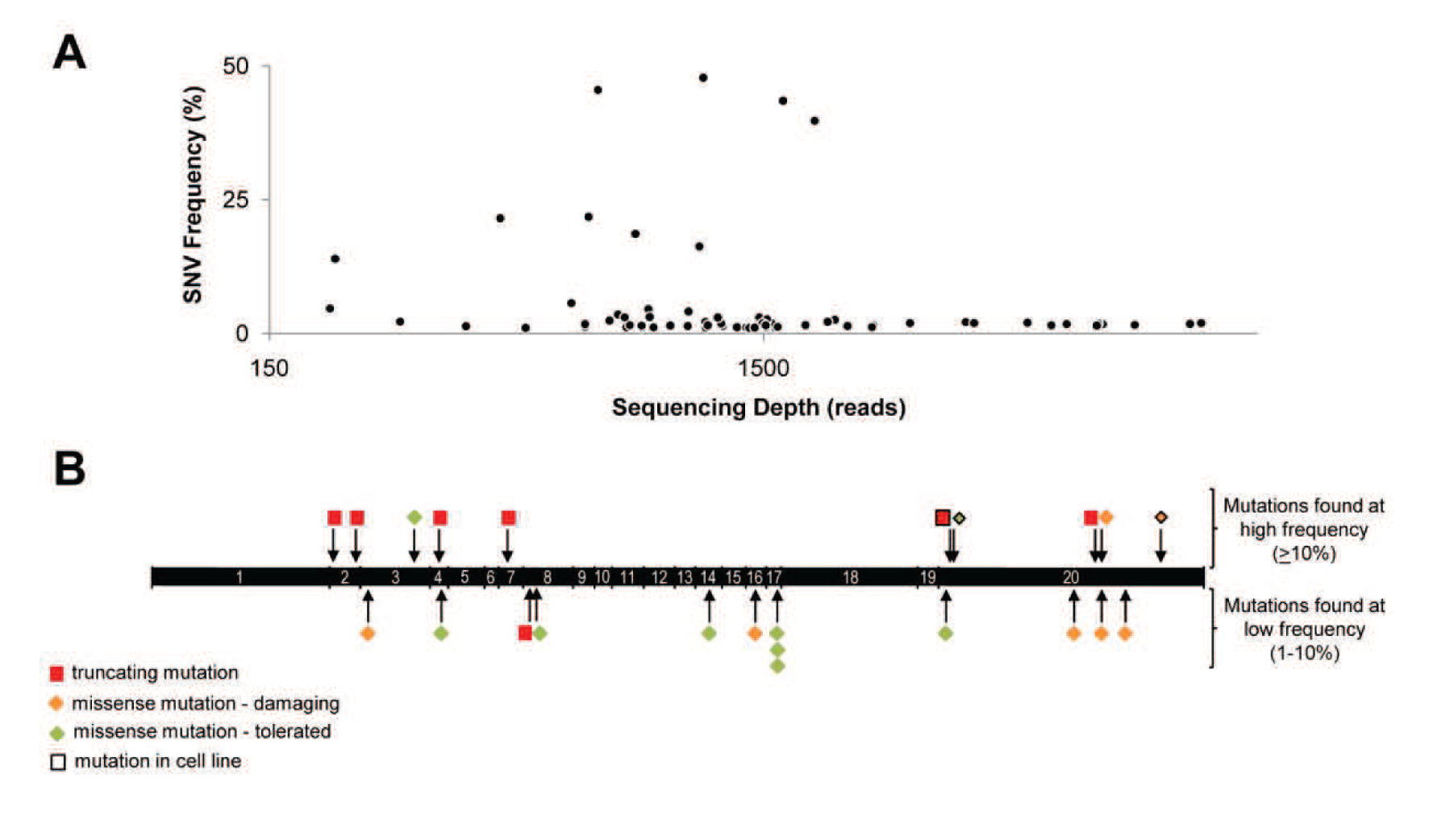

Supplement: Figure S2 — Mutation occurrence and frequency. Panel A. SNV frequency plotted against sequencing depth. Panel B. SIFT predictions for mutations comparing findings occurring for variants called at high vs. low frequency (threshold at 10%). (TIF) [file pone.0062483.s002.tif]

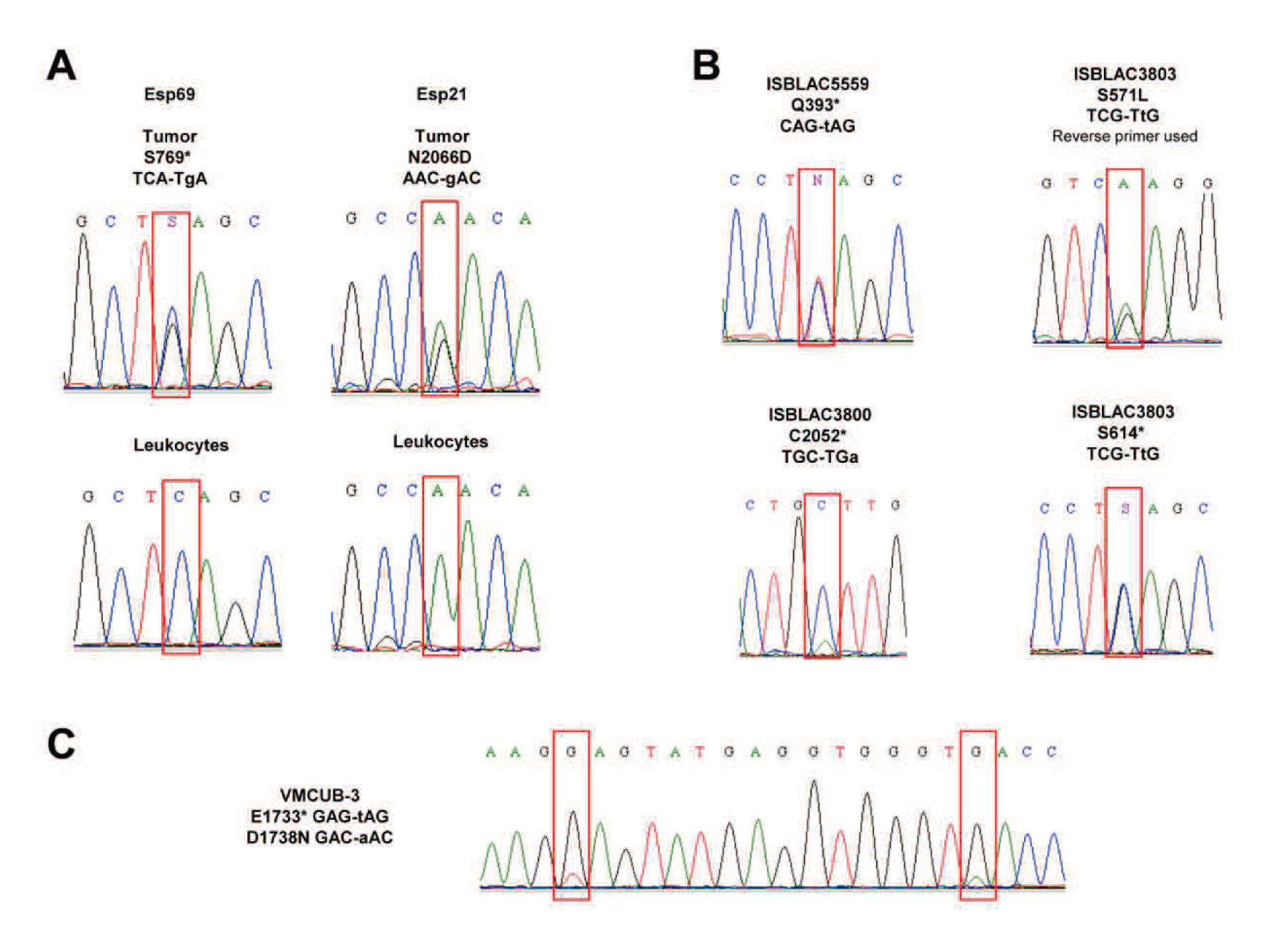

Supplement: Figure S3 — Sanger sequencing verification of all mutations detected in the resequencing study. All mutations detected at a frequency >10% were verified in both tumors and VMCUB-3 cells. The wild type sequence in normal leukocyte DNA is also shown for selected cases. (TIF) [file pone.0062483.s003.tif]

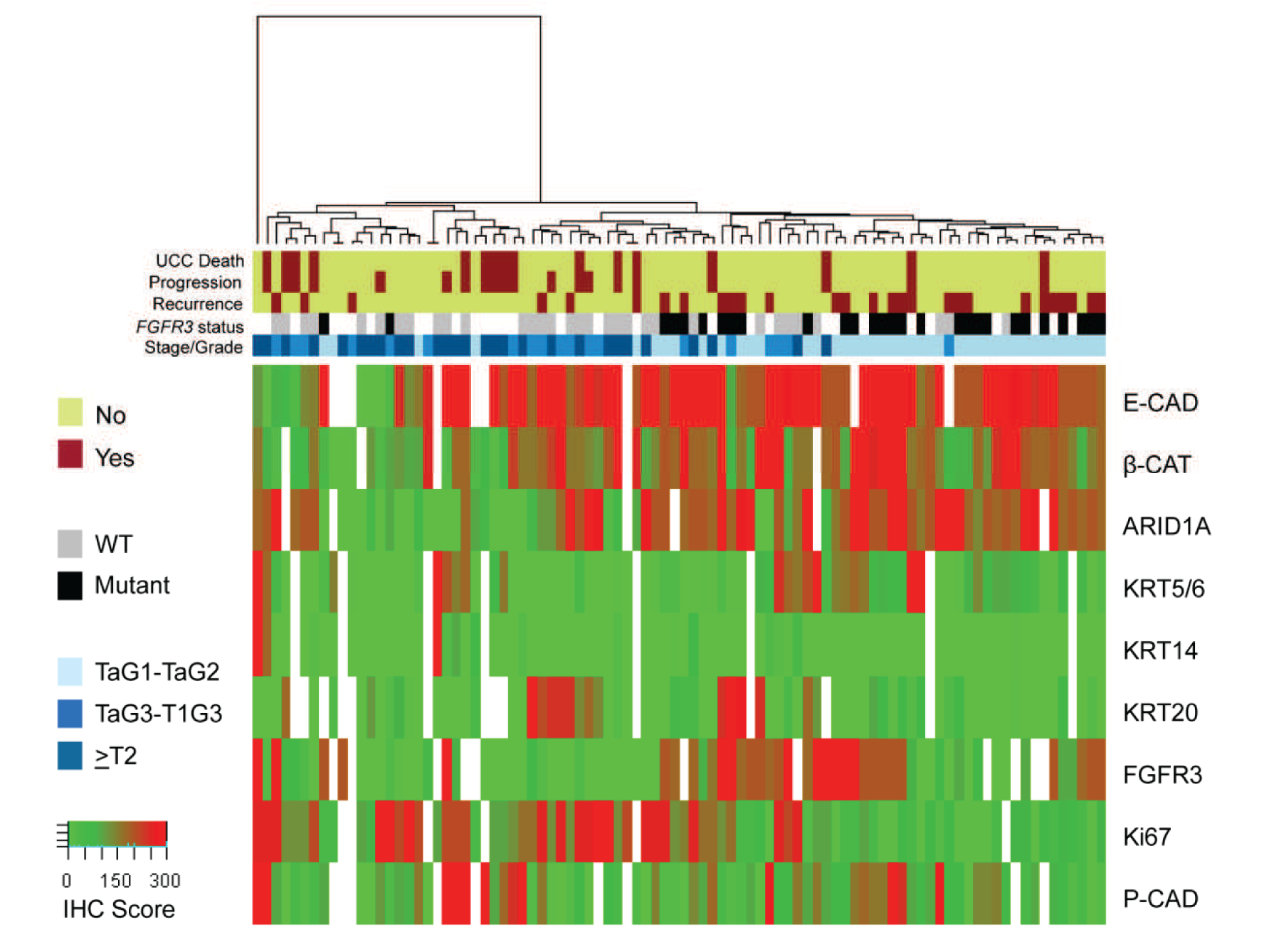

Supplement: Figure S4 — Relationship between ARID1A levels and those of other well-established UBC markers. UBC cases were classified in three categories: low grade NMI (TaG1 and TaG2 tumors), high grade NMI (TaG3 and T1G3 tumors), and MI (>T2 tumors). Nonhierarchical clustering of IHC scores for ARID1A, FGFR3, KRT5/6, KRT14, KRT20, β-CAT, Ki67, ECAD, and P-CAD was performed. IHC scores are shown using a green-red color code. Color bars under the dendogram include information about prognosis (pistachio/bourbon), tumor stage and grade (tones of blue), and FGFR3 mutational status (grey/black) when known. White squares indicate that information for that parameter is not available. (TIF) [file pone.0062483.s004.tif]

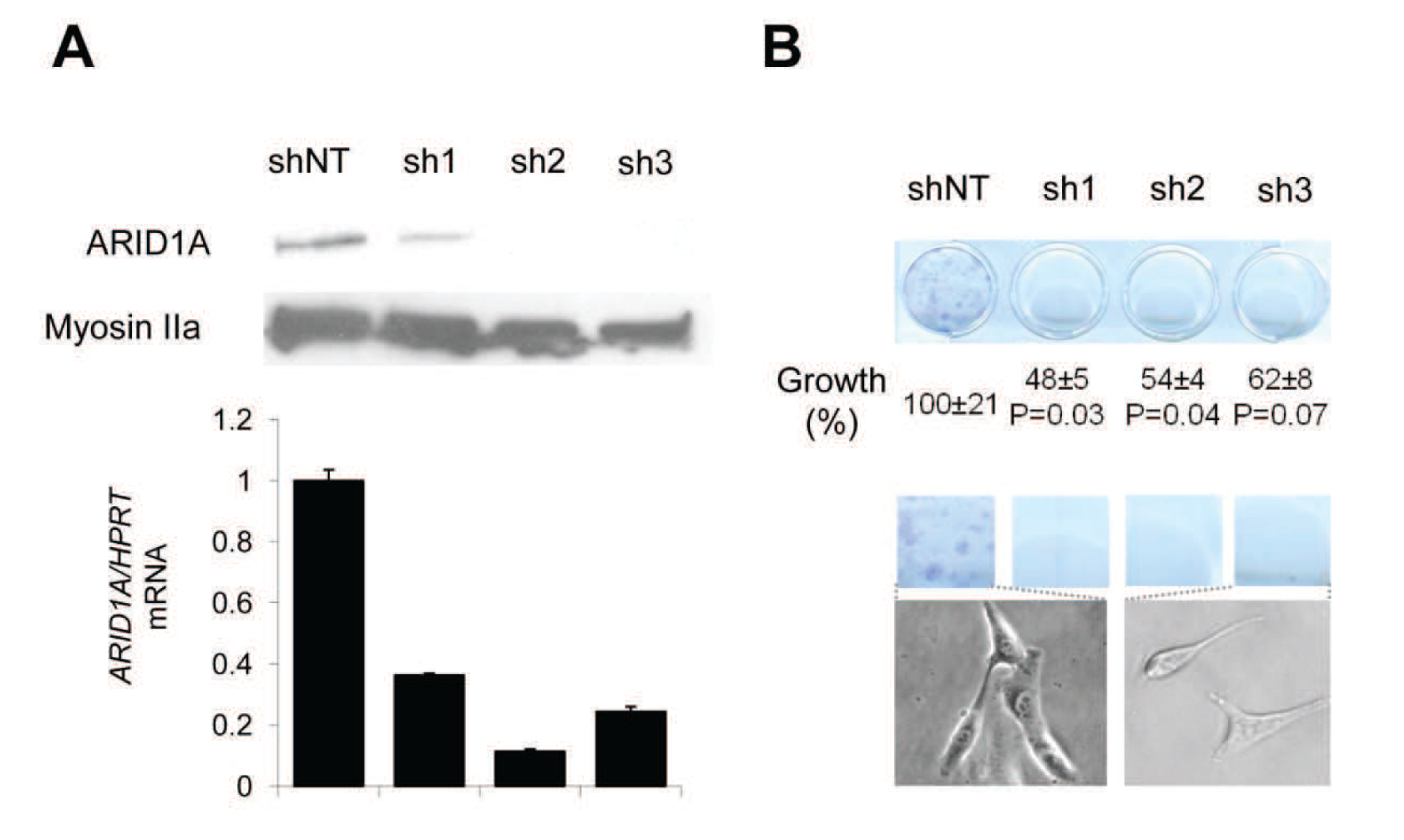

Supplement: Figure S5 — Effects of ARID1A knockdown in the 253J UBC cells. Panel A. ARID1A was knocked-down using three different shRNAs in the 253J cells. The knock-down was efficient at both the protein and mRNA levels. The bars represent the relative quantification of ARID1A mRNA levels taking non-targeting shRNA interfered cells as controls. Panel B. The quantification colony formation is shown, with error intervals of results from triplicate experiments denoting SEM. ARID1A knockdown was associated with reduced colony formation. Representative morphology changes in the cultured cells interfered for shNT (scrambled shRNA) and one of the shRNAs targeting ARID1A are shown. (TIF) [file pone.0062483.s005.tif]

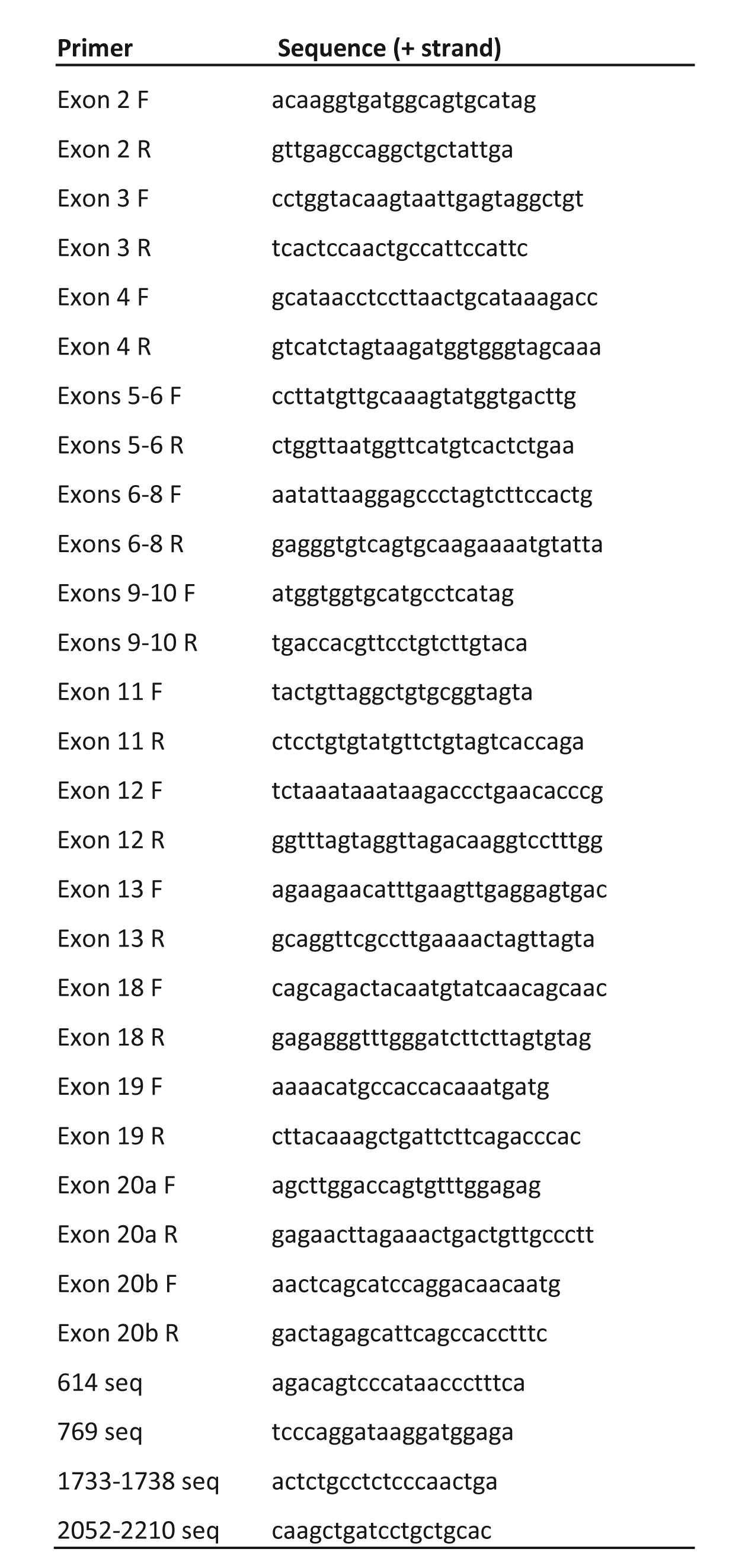

Supplement: Table S1 — List of primers used for ARID1A resequencing and Sanger sequencing. (TIF) [file pone.0062483.s006.tif]

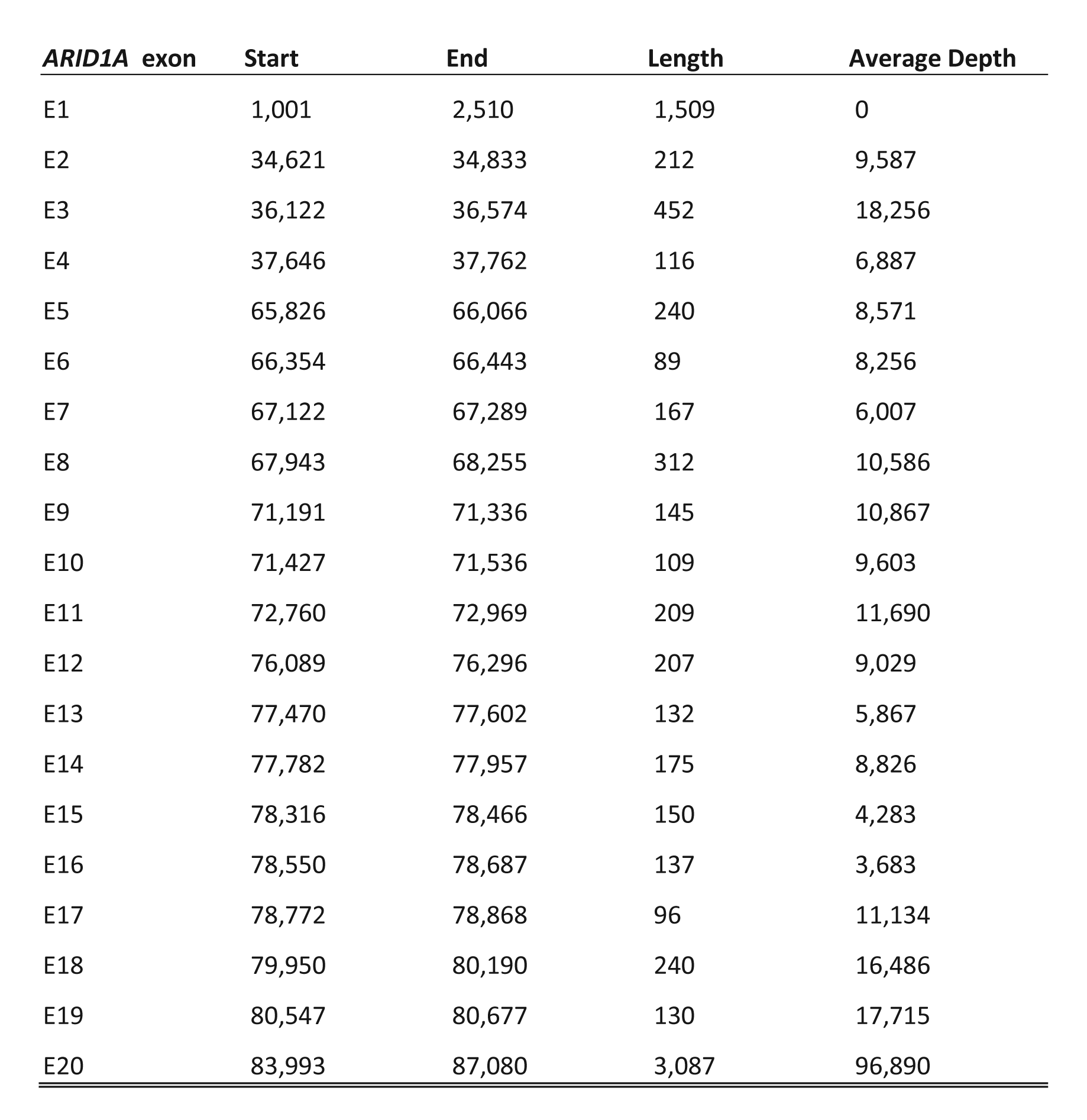

Supplement: Table S2 — Summary of reads per exon in the ARID1A resequencing study. Exon starting and ending positions are shown, along with the exon length in base pairs and the average sequencing depth. (TIF) [file pone.0062483.s007.tif]
