# Supplementary figures and images for: Correction: ARID1A Alterations Are Associated with FGFR3-Wild Type, Poor-Prognosis, Urothelial Bladder Tumors
Source: PLoS One. 2014 Jan 8;9(1):10.1371/annotation/6e83489b-e1ff-4523-9b31-08c90fa39030. doi: 10.1371/annotation/6e83489b-e1ff-4523-9b31-08c90fa39030 (PMC3888214; doi:10.1371/annotation/6e83489b-e1ff-4523-9b31-08c90fa39030)

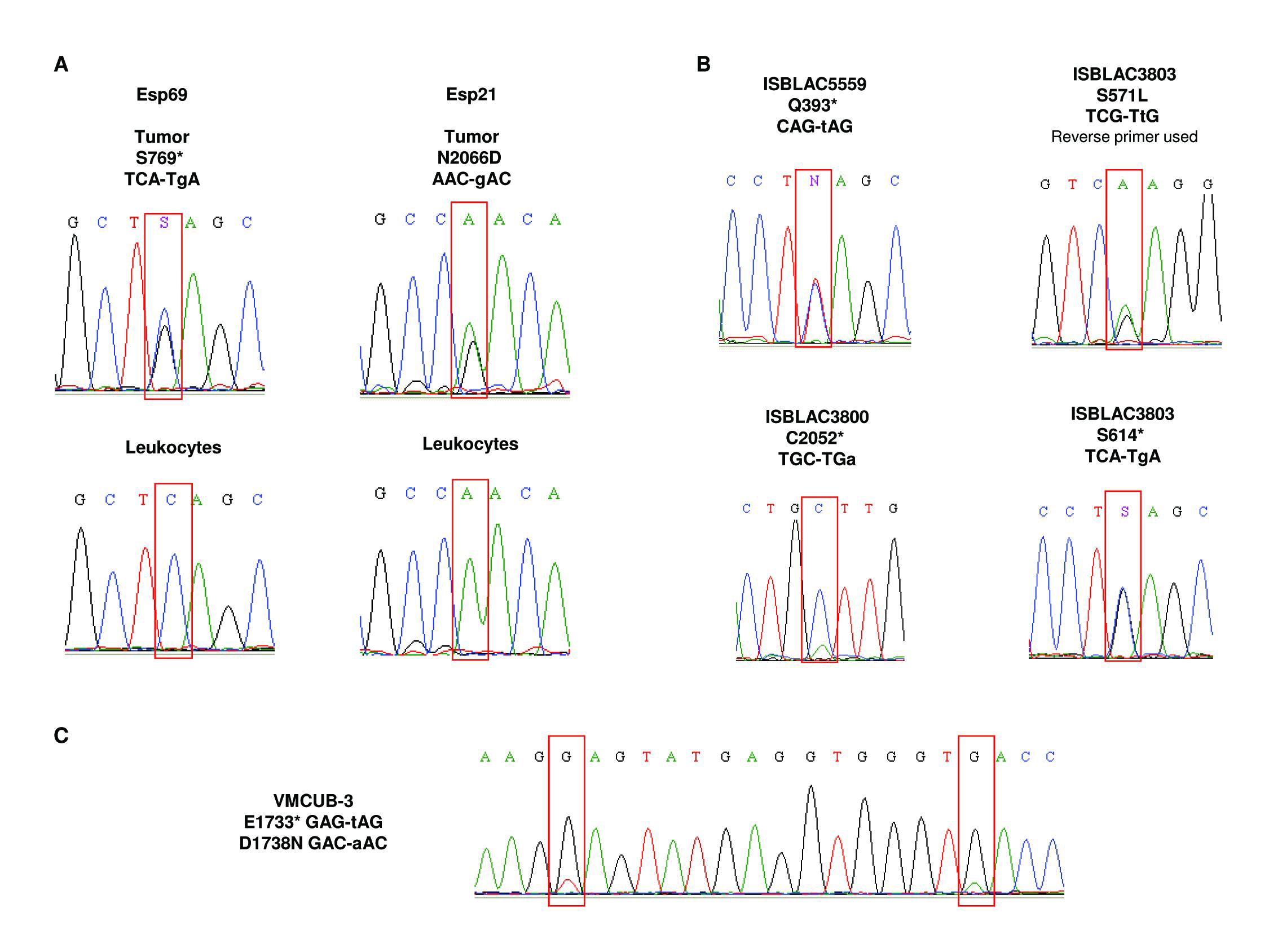

Supplement: Supplementary file 1 [file pone.6e83489b-e1ff-4523-9b31-08c90fa39030.s001.tif]
